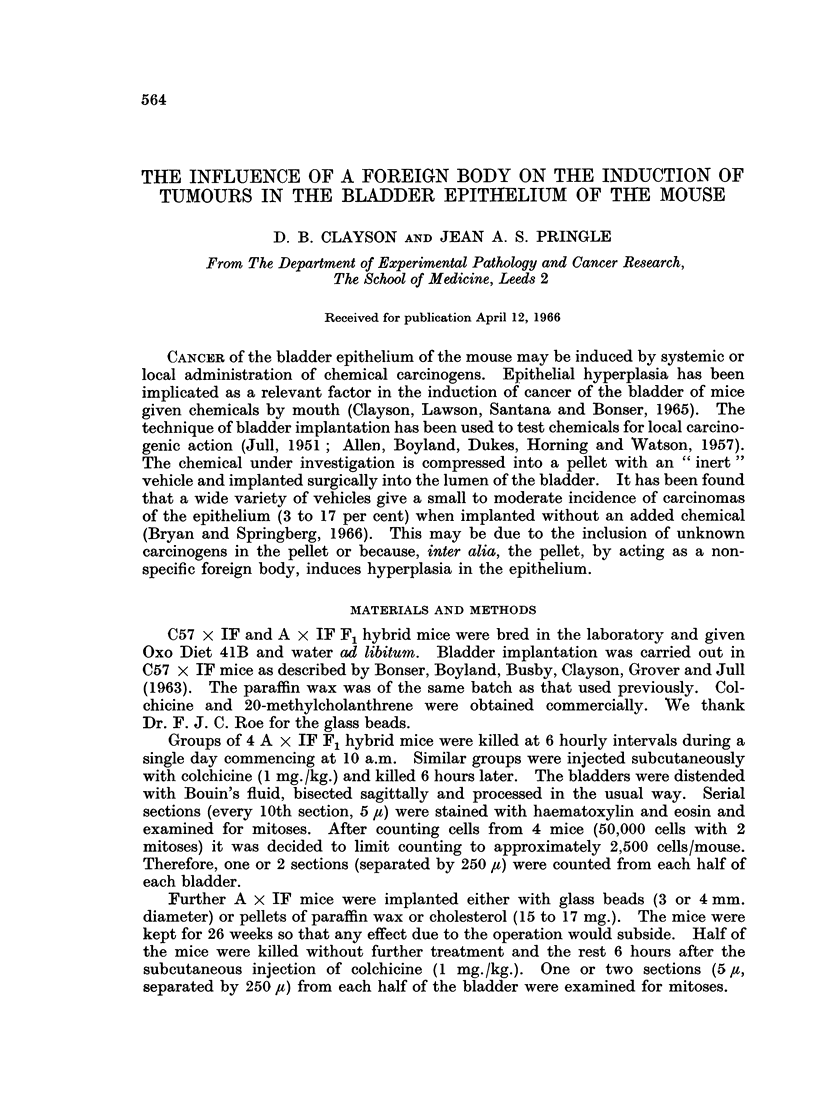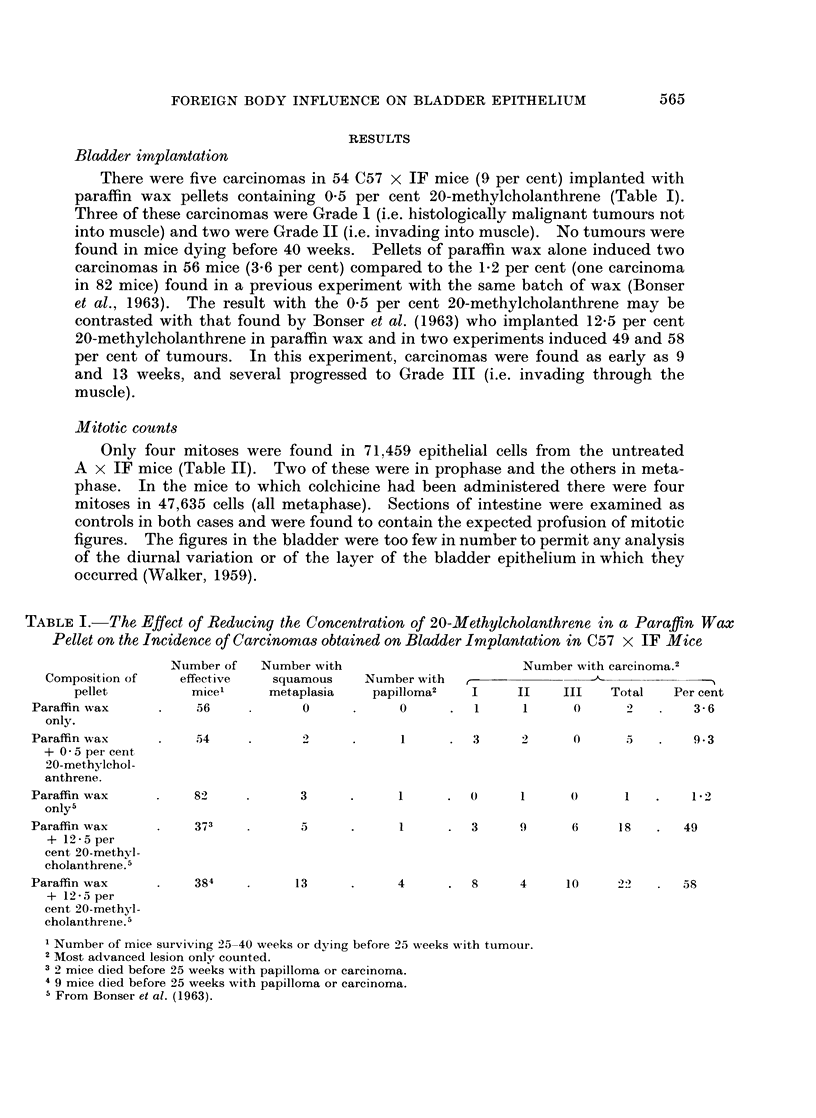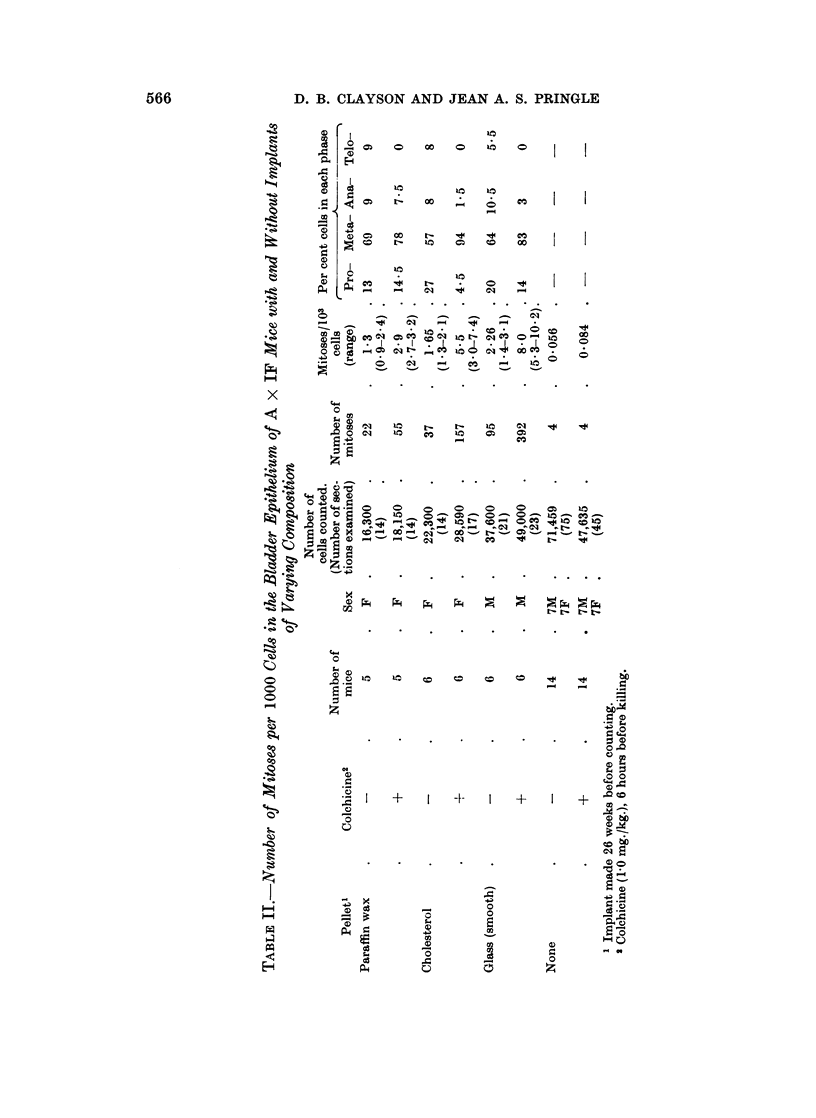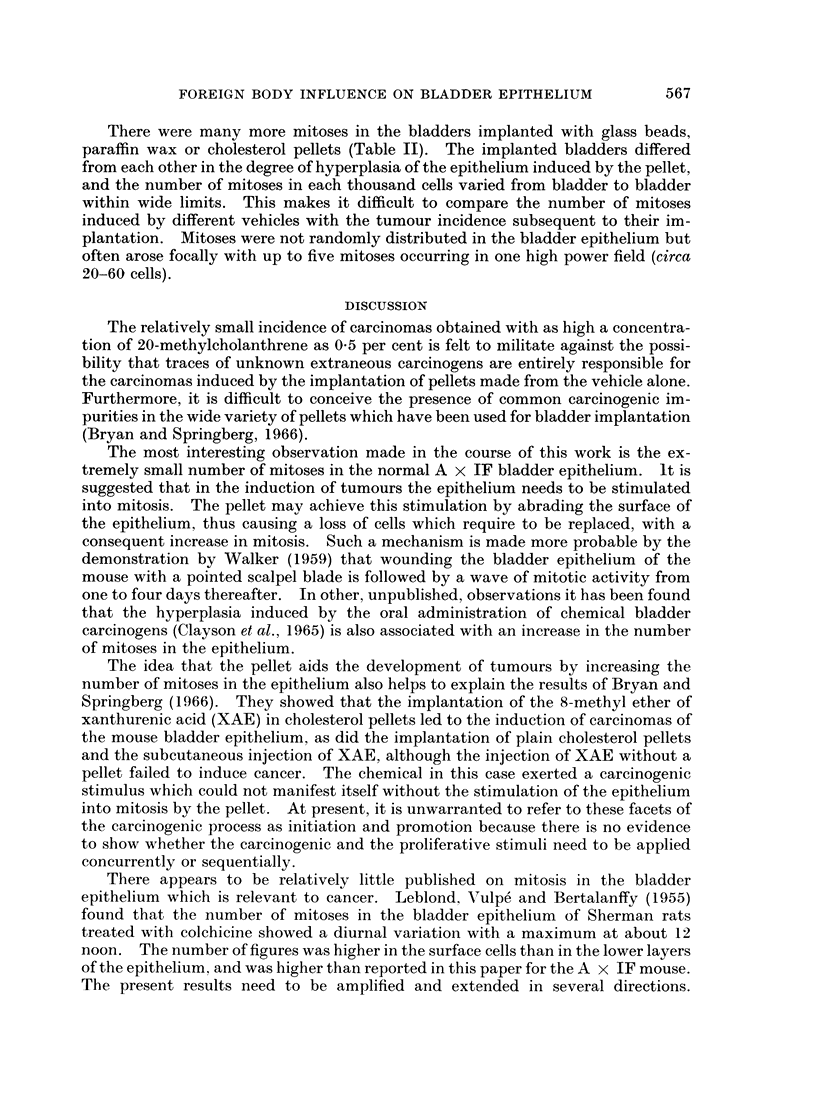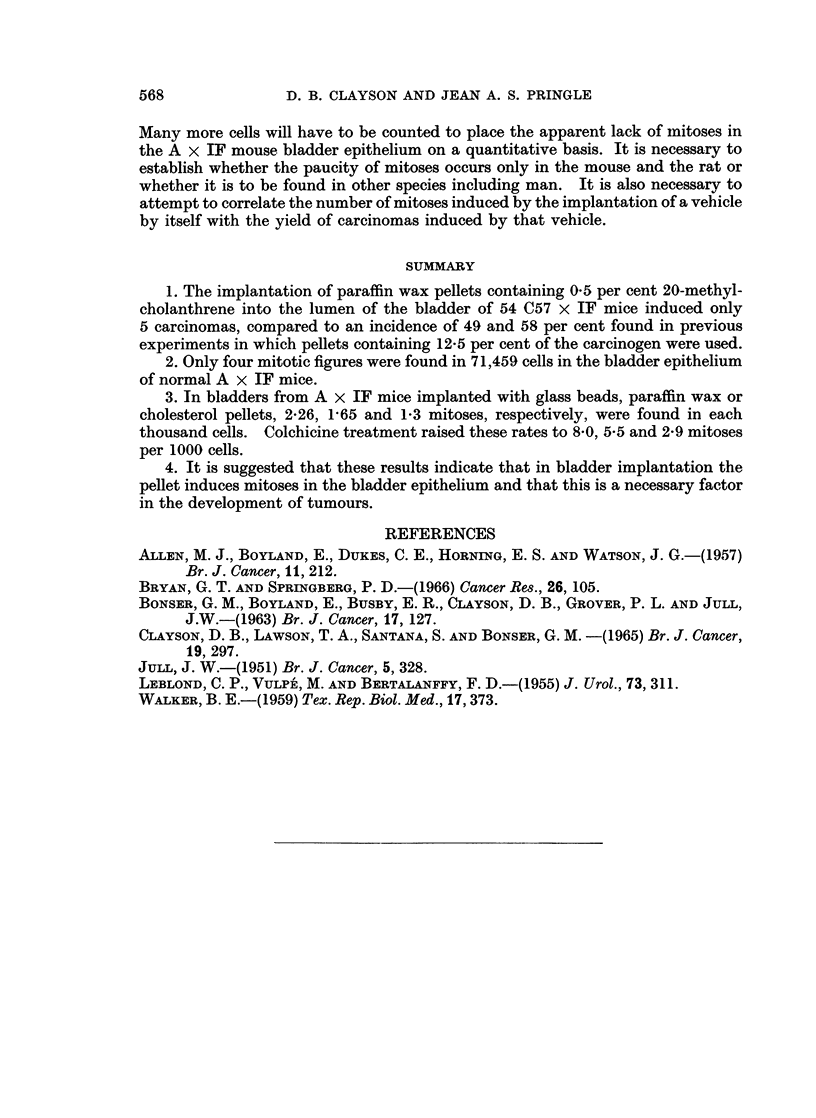# The influence of a foreign body on the induction of tumours in the bladder epithelium of the mouse.

**DOI:** 10.1038/bjc.1966.70

**Published:** 1966-09

**Authors:** D. B. Clayson, J. A. Pringle


### THE INFLUENCE OF A FOREIGN BODY ON THE INDUCTION OF TUMOURS IN THE BLADDER EPITHELIUM OF THE MOUSE

D. B. CLAYSON AND JEAN A. S. PRINGLE

*From The Department of Experimental Pathology and Cancer Research, The School of Medicine, Leeds 2*

Received for publication April 12, 1966

CANCER of the bladder epithelium of the mouse may be induced by systemic or local administration of chemical carcinogens. Epithelial hyperplasia has been implicated as a relevant factor in the induction of cancer of the bladder of mice given chemicals by mouth (Clayson, Lawson, Santana and Bonser, 1965). The technique of bladder implantation has been used to test chemicals for local carcinogenic action (Jull, 1951 ; Allen, Boyland, Dukes, Horning and Watson, 1957). The chemical under investigation is compressed into a pellet with an " inert " vehicle and implanted surgically into the lumen of the bladder. It has been found that a wide variety of vehicles give a small to moderate incidence of carcinomas of the epithelium (3 to 17 per cent) when implanted without an added chemical (Bryan and Springberg, 1966). This may be due to the inclusion of unknown carcinogens in the pellet or because, *inter alia*, the pellet, by acting as a non-specific foreign body, induces hyperplasia in the epithelium.

### MATERIALS AND METHODS

C57 × IF and A × IF $F_1$ hybrid mice were bred in the laboratory and given Oxo Diet 41B and water *ad libitum*. Bladder implantation was carried out in C57 × IF mice as described by Bonser, Boyland, Busby, Clayson, Grover and Jull (1963). The paraffin wax was of the same batch as that used previously. Colchicine and 20-methylcholanthrene were obtained commercially. We thank Dr. F. J. C. Roe for the glass beads.

Groups of 4 A × IF $F_1$ hybrid mice were killed at 6 hourly intervals during a single day commencing at 10 a.m. Similar groups were injected subcutaneously with colchicine (1 mg./kg.) and killed 6 hours later. The bladders were distended with Bouin's fluid, bisected sagittally and processed in the usual way. Serial sections (every 10th section, 5 $\mu$) were stained with haematoxylin and eosin and examined for mitoses. After counting cells from 4 mice (50,000 cells with 2 mitoses) it was decided to limit counting to approximately 2,500 cells/mouse. Therefore, one or 2 sections (separated by 250 $\mu$) were counted from each half of each bladder.

Further A × IF mice were implanted either with glass beads (3 or 4 mm. diameter) or pellets of paraffin wax or cholesterol (15 to 17 mg.). The mice were kept for 26 weeks so that any effect due to the operation would subside. Half of the mice were killed without further treatment and the rest 6 hours after the subcutaneous injection of colchicine (1 mg./kg.). One or two sections (5 $\mu$, separated by 250 $\mu$) from each half of the bladder were examined for mitoses.

### RESULTS

*Bladder implantation*

There were five carcinomas in 54 C57 × IF mice (9 per cent) implanted with paraffin wax pellets containing 0·5 per cent 20-methylcholanthrene (Table I). Three of these carcinomas were Grade I (i.e. histologically malignant tumours not into muscle) and two were Grade II (i.e. invading into muscle). No tumours were found in mice dying before 40 weeks. Pellets of paraffin wax alone induced two carcinomas in 56 mice (3·6 per cent) compared to the 1·2 per cent (one carcinoma in 82 mice) found in a previous experiment with the same batch of wax (Bonser *et al.*, 1963). The result with the 0·5 per cent 20-methylcholanthrene may be contrasted with that found by Bonser *et al.* (1963) who implanted 12·5 per cent 20-methylcholanthrene in paraffin wax and in two experiments induced 49 and 58 per cent of tumours. In this experiment, carcinomas were found as early as 9 and 13 weeks, and several progressed to Grade III (i.e. invading through the muscle).

*Mitotic counts*

Only four mitoses were found in 71,459 epithelial cells from the untreated A × IF mice (Table II). Two of these were in prophase and the others in metaphase. In the mice to which colchicine had been administered there were four mitoses in 47,635 cells (all metaphase). Sections of intestine were examined as controls in both cases and were found to contain the expected profusion of mitotic figures. The figures in the bladder were too few in number to permit any analysis of the diurnal variation or of the layer of the bladder epithelium in which they occurred (Walker, 1959).

TABLE I.—*The Effect of Reducing the Concentration of* 20-*Methylcholanthrene in a Paraffin Wax Pellet on the Incidence of Carcinomas obtained on Bladder Implantation in* C57 × IF *Mice*

| Composition of pellet | Number of effective mice[1] | Number with squamous metaplasia | Number with papilloma[2] | Number with carcinoma.[2] | | | | |
|---|---|---|---|---|---|---|---|---|
| | | | | I | II | III | Total | Per cent |
| Paraffin wax only. | 56 | 0 | 0 | 1 | 1 | 0 | 2 | 3·6 |
| Paraffin wax + 0·5 per cent 20-methylcholanthrene. | 54 | 2 | 1 | 3 | 2 | 0 | 5 | 9·3 |
| Paraffin wax only[5] | 82 | 3 | 1 | 0 | 1 | 0 | 1 | 1·2 |
| Paraffin wax + 12·5 per cent 20-methylcholanthrene.[5] | 37[3] | 5 | 1 | 3 | 9 | 6 | 18 | 49 |
| Paraffin wax + 12·5 per cent 20-methylcholanthrene.[5] | 38[4] | 13 | 4 | 8 | 4 | 10 | 22 | 58 |

[1] Number of mice surviving 25–40 weeks or dying before 25 weeks with tumour.
[2] Most advanced lesion only counted.
[3] 2 mice died before 25 weeks with papilloma or carcinoma.
[4] 9 mice died before 25 weeks with papilloma or carcinoma.
[5] From Bonser *et al.* (1963).

TABLE II.—*Number of Mitoses per* 1000 *Cells in the Bladder Epithelium of* A × IF *Mice with and Without Implants of Varying Composition*

| Pellet[1] | Colchicine[2] | Number of mice | Sex | Number of cells counted. (Number of sections examined) | Number of mitoses | Mitoses/$10^3$ cells (range) | Per cent cells in each phase: Pro– | Meta– | Ana– | Telo– |
|---|---|---|---|---|---|---|---|---|---|---|
| Paraffin wax | – | 5 | F | 16,300 (14) | 22 | 1·3 (0·9–2·4) | 13 | 69 | 9 | 9 |
| | + | 5 | F | 18,150 (14) | 55 | 2·9 (2·7–3·2) | 14·5 | 78 | 7·5 | 0 |
| Cholesterol | – | 6 | F | 22,300 (14) | 37 | 1·65 (1·3–2·1) | 27 | 57 | 8 | 8 |
| | + | 6 | F | 28,590 (17) | 157 | 5·5 (3·0–7·4) | 4·5 | 94 | 1·5 | 0 |
| Glass (smooth) | – | 6 | M | 37,600 (21) | 95 | 2·26 (1·4–3·1) | 20 | 64 | 10·5 | 5·5 |
| | + | 6 | M | 49,000 (23) | 392 | 8·0 (5·3–10·2) | 14 | 83 | 3 | 0 |
| None | – | 14 | 7M 7F | 71,459 (75) | 4 | 0·056 | — | — | — | — |
| | + | 14 | 7M 7F | 47,635 (45) | 4 | 0·084 | — | — | — | — |

[1] Implant made 26 weeks before counting.
[2] Colchicine (1·0 mg./kg.), 6 hours before killing.

There were many more mitoses in the bladders implanted with glass beads, paraffin wax or cholesterol pellets (Table II). The implanted bladders differed from each other in the degree of hyperplasia of the epithelium induced by the pellet, and the number of mitoses in each thousand cells varied from bladder to bladder within wide limits. This makes it difficult to compare the number of mitoses induced by different vehicles with the tumour incidence subsequent to their implantation. Mitoses were not randomly distributed in the bladder epithelium but often arose focally with up to five mitoses occurring in one high power field (*circa* 20–60 cells).

### DISCUSSION

The relatively small incidence of carcinomas obtained with as high a concentration of 20-methylcholanthrene as 0·5 per cent is felt to militate against the possibility that traces of unknown extraneous carcinogens are entirely responsible for the carcinomas induced by the implantation of pellets made from the vehicle alone. Furthermore, it is difficult to conceive the presence of common carcinogenic impurities in the wide variety of pellets which have been used for bladder implantation (Bryan and Springberg, 1966).

The most interesting observation made in the course of this work is the extremely small number of mitoses in the normal A × IF bladder epithelium. It is suggested that in the induction of tumours the epithelium needs to be stimulated into mitosis. The pellet may achieve this stimulation by abrading the surface of the epithelium, thus causing a loss of cells which require to be replaced, with a consequent increase in mitosis. Such a mechanism is made more probable by the demonstration by Walker (1959) that wounding the bladder epithelium of the mouse with a pointed scalpel blade is followed by a wave of mitotic activity from one to four days thereafter. In other, unpublished, observations it has been found that the hyperplasia induced by the oral administration of chemical bladder carcinogens (Clayson *et al.*, 1965) is also associated with an increase in the number of mitoses in the epithelium.

The idea that the pellet aids the development of tumours by increasing the number of mitoses in the epithelium also helps to explain the results of Bryan and Springberg (1966). They showed that the implantation of the 8-methyl ether of xanthurenic acid (XAE) in cholesterol pellets led to the induction of carcinomas of the mouse bladder epithelium, as did the implantation of plain cholesterol pellets and the subcutaneous injection of XAE, although the injection of XAE without a pellet failed to induce cancer. The chemical in this case exerted a carcinogenic stimulus which could not manifest itself without the stimulation of the epithelium into mitosis by the pellet. At present, it is unwarranted to refer to these facets of the carcinogenic process as initiation and promotion because there is no evidence to show whether the carcinogenic and the proliferative stimuli need to be applied concurrently or sequentially.

There appears to be relatively little published on mitosis in the bladder epithelium which is relevant to cancer. Leblond, Vulpé and Bertalanffy (1955) found that the number of mitoses in the bladder epithelium of Sherman rats treated with colchicine showed a diurnal variation with a maximum at about 12 noon. The number of figures was higher in the surface cells than in the lower layers of the epithelium, and was higher than reported in this paper for the A × IF mouse. The present results need to be amplified and extended in several directions.

Many more cells will have to be counted to place the apparent lack of mitoses in the A × IF mouse bladder epithelium on a quantitative basis. It is necessary to establish whether the paucity of mitoses occurs only in the mouse and the rat or whether it is to be found in other species including man. It is also necessary to attempt to correlate the number of mitoses induced by the implantation of a vehicle by itself with the yield of carcinomas induced by that vehicle.

### SUMMARY

1. The implantation of paraffin wax pellets containing 0·5 per cent 20-methylcholanthrene into the lumen of the bladder of 54 C57 × IF mice induced only 5 carcinomas, compared to an incidence of 49 and 58 per cent found in previous experiments in which pellets containing 12·5 per cent of the carcinogen were used.

2. Only four mitotic figures were found in 71,459 cells in the bladder epithelium of normal A × IF mice.

3. In bladders from A × IF mice implanted with glass beads, paraffin wax or cholesterol pellets, 2·26, 1·65 and 1·3 mitoses, respectively, were found in each thousand cells. Colchicine treatment raised these rates to 8·0, 5·5 and 2·9 mitoses per 1000 cells.

4. It is suggested that these results indicate that in bladder implantation the pellet induces mitoses in the bladder epithelium and that this is a necessary factor in the development of tumours.

### REFERENCES

Allen, M. J., Boyland, E., Dukes, C. E., Horning, E. S. and Watson, J. G.—(1957) *Br. J. Cancer*, **11**, 212.

Bryan, G. T. and Springberg, P. D.—(1966) *Cancer Res.*, **26**, 105.

Bonser, G. M., Boyland, E., Busby, E. R., Clayson, D. B., Grover, P. L. and Jull, J.W.—(1963) *Br. J. Cancer*, **17**, 127.

Clayson, D. B., Lawson, T. A., Santana, S. and Bonser, G. M. —(1965) *Br. J. Cancer*, **19**, 297.

Jull, J. W.—(1951) *Br. J. Cancer*, **5**, 328.

Leblond, C. P., Vulpé, M. and Bertalanffy, F. D.—(1955) *J. Urol.*, **73**, 311.

Walker, B. E.—(1959) *Tex. Rep. Biol. Med.*, **17**, 373.